# Plasma proenkephalin and neutrophil gelatinase-associated lipocalin predict mortality in ICU patients with acute kidney injury

**DOI:** 10.1186/s12882-024-03611-0

**Published:** 2024-05-22

**Authors:** Mengqin Zhang, Yang Yang, Luqi Zhu, Ke Cui, Sheng Zhang, Yinghe Xu, Yongpo Jiang

**Affiliations:** 1 Department of Critical Care Medicine, Taizhou Hospital of Zhejiang Province Affiliated to Wenzhou Medical University, No. 150, XiMen Street, Taizhou, China; 2 Department of Orthopedics, Taizhou Hospital of Zhejiang Province Affiliated to Wenzhou Medical University, Taizhou, China

**Keywords:** Acute kidney injury, Intensive care unit, Mortality, Neutrophil gelatinase-associated lipocalin, Prognosis, Proenkephalin

## Abstract

**Background:**

Acute kidney injury (AKI) is a common complication in patients admitted to intensive care unit (ICU) and mortality rates for this condition are high. To reduce the high incidence of short-term mortality, reliable prognostic indicators are required to facilitate early diagnosis and treatment of AKI. We assessed the ability of plasma proenkephalin (p‑PENK) and plasma neutrophil gelatinase-associated lipocalin (p‑NGAL) to predict 28-day mortality in AKI patients in intensive care.

**Methods:**

This prospective study, carried out between January 2019 and December 2019, comprised 150 patients (100 male) diagnosed with AKI after excluding 20 patients discharged within 24 h and those with missing hospitalization data. Blood samples were collected to determine admission p-PENK and p-NGAL levels. The study outcome was 28‑day mortality.

**Results:**

The mean patient age was 68 years (female, 33%). The average P‑PENK and p‑NGAL levels were 0.24 ng/µL and 223.70 ng/mL, respectively. P‑PENK levels >0.36 ng/µL and p‑NGAL levels >230.30 ng/mL were used as critical values to reliably indicate 28‑day mortality for patients with AKI (adjusted hazard ratios 0.785 [95% confidence interval 0.706–0.865, *P*<0.001] and 0.700 [95% confidence interval 0.611–0.789, *P*<0.001], respectively). This association was significant for mortality in patients in intensive care with AKI. Baseline p-PENK (0.36 ng/µL) and p-NGAL (230.30 ng/mL) levels and their respective cut-off values showed clinical value in predicting 28-day mortality.

**Conclusion:**

Serum PENK and NGAL levels, when used in conjunction, improved the accuracy of predicting 28-day mortality in patients with AKI while retaining sensitivity and specificity.

## Introduction

The incidence of acute kidney injury (AKI) in the intensive care unit (ICU) is the highest among all hospital departments, at >50%. Patients in the ICU with AKI have serious outcomes, which incur high levels of healthcare expenditure [1]. The current gold standard for AKI diagnosis is based on the consensus definitions of Kidney Disease: Improving Global Outcomes (KDIGO), which focuses on AKI stage. The higher the stage, the higher the mortality rate. The risk of death at stage 3 (47.8 %) has been reported to be significantly higher than that at Stages 2 (28.5 %) and l (15.9%); however, AKI stage is closely associated with basal renal function [2]. As most patients admitted to the ICU have unclear baseline renal function and complex conditions (combined with disruption of the acid-base balance, electrolyte levels, fluid regulation; the accumulation of uremic toxins; and multiple organ insufficiency), it is often challenging to predict patient outcomes based on AKI grade. Therefore, new biomarkers with high sensitivity and specificity that are unaffected by a patient’s underlying renal function and current disease complexity need to be determined to address unmet medical demands.

Proenkephalin (PENK) is a stable, non-protein-binding, low-interfering, small endogenous opioid peptide with a long half-life, produced by nephron structures in response to damage [3]. It is harmful to renal function, [4] and has also been reported to have a strong negative correlation with estimated glomerular filtration rate (eGFR) [5]. Neutrophil gelatinase-associated lipocalin (NGAL) is a frequently studied biomarker for predicting AKI [6]. NGAL is produced by distal tubular cells in response to injury and combines with iron to form siderophores [7]. It protects tubular epithelial cells from cell death by upregulating heme oxygenase-1. This complex converts renal progenitors into epithelial tubules. Animal experiments have shown that NGAL protects against AKI by activating autophagy and inhibiting apoptosis [8, 9].

Our goal is to more accurately assess the prognoses of patients with AKI in intensive care units using commonly tested biomarkers at the time, without considering their previous renal function statuses and current renal function grades, as well as the complexities of their current clinical diseases.

## Methods

This study is a prospective cohort study and was reviewed and approved by the Ethics Committee of Taizhou Hospital of Zhejiang Province with approval number K20190102. Consecutive patients from Taizhou Hospital of Zhejiang Province who were admitted to the ICU at our hospital between January 2019 and December 2019 were included. Written informed consent was obtained from the patient or next of kin if the patient was unable to provide consent on admission.

Inclusion criteria comprised patients (i) who had AKI was defined on admission to the ICU according to KDIGO criteria [10]: Stage 1: serum creatinine increased 1.5 times baseline or >0.3mg/dl (26.5 µmol/l) or urinary output< 0.5ml/kg/h during a 6-hour block. Stage 2: serum creatinine increase 2.0–2.9 times baseline or urinary output <0.5ml/kg/h during two 6 hour blocks. Stage 3: Serum creatinine increase >3 times baseline or serum creatinine increase >3 times baseline or initiation of renal replacement therapy or urinary output <0.3ml/kg/h during more than 24 hours or anuria for more than 12 hours.Exclusion criteria comprised patients (i) who were admitted to ICU or who had died within 24 h post-admission to the hospital, (ii) had stage V chronic kidney disease (CKD) according to KDIGO (eGFR <15 mL/min/1.73 m^2^), and prior histories of dialysis. (iii) who were pregnant women.

Data concerning patients’ medical histories, laboratory test results, and 28-day mortality rates were analyzed. All samples were collected in the ICU within 24 hours directly after the patients had been diagnosed with AKI and admitted to the ICU. Blood samples from the patients in the study were collected. Each blood sample was then centrifuged, and the isolated plasma was immediately stored at -80°C. Enzyme-linked immunosorbent assay (ELISA; SED396Hu 96T) was used to determine P-PENK concentrations. ELISA (QuicKey Human NGAL ELISA Kit, E-TSEL-H0003 96T/48T/24T) was also used to determine P-NGAL concentrations. Hemoglobin(HGB) , leukocyte, albumin, alanine aminotransferase, creatinine, urea nitrogen (BUN), serum calcium ions levels, and arterial blood gas (including serum potassium and lactate levels) were analyzed at local laboratories. AKI was defined according to KDIGO criteria [10]. The main outcome in this study was 28-day all-cause mortality. The vital statuses of patients during follow-up were determined through direct contact with the patient. The local ethics committee approved the study, which was conducted in accordance with the Declaration of Helsinki.

## Statistical analysis

Age, body mass index(BMI), HGB, Leukocytes, Albumin, BUN, Ca^2+^, K^+,^ and Lactate are shown (Table 1) as the mean ± standard deviation. Acute physiology and chronic health evaluation (APACHE) II score, the sequential organ failure assessment (SOFA) score, Creatinine, alanine aminotransferase (ALT) , p-PENK and p-NGAL are shown (Table 1) as interquartile range (IQR). Gender is presented as number (n). Comorbidities such as hypertension, diabetes, cardiovascular disease, vasoactive agents, and glucocorticoids are presented (Table 1) as percentages (%). Independent t-tests were used for the normally distributed data. Fisher’s exact test was used for categorical variables. Mann–Whitney U or Student’s t-tests were used for continuous variables. Analysis of variance was used for the non-positive distribution. Kaplan–Meier survival analysis was used to estimate the differences in survival between groups. A Multivariate Cox proportional hazard regression model was used to assess the prognostic values of the variables. Factors with *P* < 0.05 and those deemed clinically significant were included in the multivariate analysis.The receiver operating-characteristic curve (ROC) analysis was used to predict mortality. The predictive power of p-PENK and p-NGAL was assessed using the area under the receiver operating characteristic curve (AUROC). The Younden index was used for determining cut-off points.The cut-off value for statistical differences was considered to be *P* < 0.05. SPSS 22.0 (IBM Corp, Armonk, NY, USA) was used to analyze the data.

**Table 1:** Data of critically ill patients on admission

	ALL (*n*=150)	28-day survive group (*n*=107)	28-day mortality group (*n*=43)	*p*-value
Gender (male/female)	100/50	70/37	30/13	0.610
Age(year); mean±SD	68.39±15.31	67.33±14.86	71.02±16.27	0.182
BMI; mean±SD	22.66±2.64	22.97±2.66	21.90±2.45	0.024
HGB( hemoglobin ); mean±SD	109.45±26.28	110.07±26.41	107.93±26.19	0.654
Leukocytes (10E9); mean±SD	13.15±7.18	13.47±7.44	12.38±6.51	0.402
Albumin(g/L); mean±SD	28.65±5.51	29.23±5.50	27.21±5.31	0.041
ALT(glutamic-pyruvic transaminase); median(IQR)	39.00(19.00~116.25)	35.00(18.00~102.00)	70.00(20.10~273.00)	0.084
Creatinine(umol/L); median(IQR)	160.50(119.50~256.00)	156.00(111.00~236.00)	176.00(129.00~286.00)	0.888
BUN(mmol/L); mean±SD	15.73±7.47	15.14±7.74	17.19±6.64	0.128
Ca2+(mmol/L);mean±SD	1.60±0.46	1.58±0.46	1.65±0.44	0.375
K^+^(mmol/L);mean±SD	4.29±0.75	4.25±0.77	4.37±0.71	0.387
Lactate(mmol/L); mean±SD	2.79±2.73	2.46±2.66	3.61±2.76	0.020
Hypertension; *n*=(%)	80(53.33%)	59(55.14%)	21(48.84%)	0.484
Diabitus; *n*=(%)	39(26.00%)	27(25.23%)	12(27.91%)	0.736
Cardiovascular disease; *n*=(%)	18(12.00%)	14(13.08%)	4(9.30%)	0.519
Apache II score; median(IQR)	21(16.75~25.00)	21(17~25)	23(16~27)	0.239
SOFA score; median(IQR)	9(6~14)	9(6~13)	12(6~17)	0.029
Vasoactive agents; *n*=(%)	61(40.67%)	39(36.45%)	22(51.16%)	0.097
Glucocorticoid; *n*=(%)	14(9.33)	8(4.48%)	6(13.95%)	0.227
AKI stage				
Stage1 *n*=(%)	66(44%)	53(49.53%)	13(30.23%)	0.001
Stage2 *n*=(%)	27(18%)	23(21.5%)	4(9.3%)	
Stage3 *n*=(%)	57(38%)	31(28.97%)	26(60.47%)	
Source of admission				
Sepsis or sepsis shock( *n*=(%))	64(42.67%)	44(41.12%)	20(46.51%)	0.498
Cardiac ( *n*=(%))	52(34.67%)	37(34.58%)	15(34.88%)	
Noncardiac surgery( *n*=(%))	20(13.33%)	17(15.89%)	3(6.98%)	
Others( *n*=(%))	14(9.33%)	9(8.41%)	5(11.63%)	
p-PENK(ng/ul) median(IQR)	0.24(0.16~0.47)	0.20(0.14~0.34)	0.51(0.29~0.62)	<0.001
p-NGAL(ng/ml) median(IQR)	223.70(155.42~290.47)	207.53(135.10~265.01)	265.59(216.91~332.85)	<0.001

## Results

We included 150 patients (men,* n* = 100) in our study (Table 1). The most important reason for rejection was automatic discharge within 24 h after admission to the ICU (12 patients, 60% of the excluded group), followed by a lack of data on hospitalization (seven patients, 35% of the excluded group) and comorbid end-stage renal disease (one patient, 5% of the excluded group).

Patient baseline characteristics (*n* = 150) stratified according to 28-day mortality are shown in Table 1. The mean age was 68 years, and 67% of the patients in the study were men (Table 1). The overall 28-day mortality was 28.7%. The median p-PENK and p-NGAL values were 0.24 ng/µL (IQR 0.16–0.47) and 223.70 ng/mL (IQR 155.42–290.47), respectively (Table 1) .

**Fig. 1:**
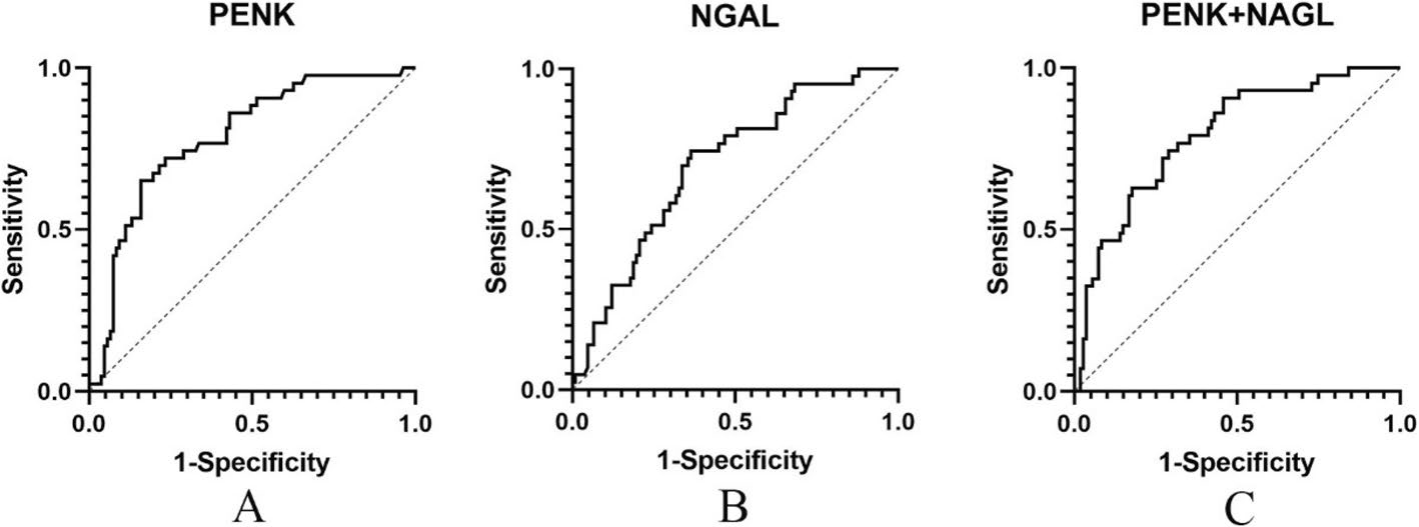
ROC curves of p-PENK and p-NGAL to predict 28-day mortality

**Fig. 2:**
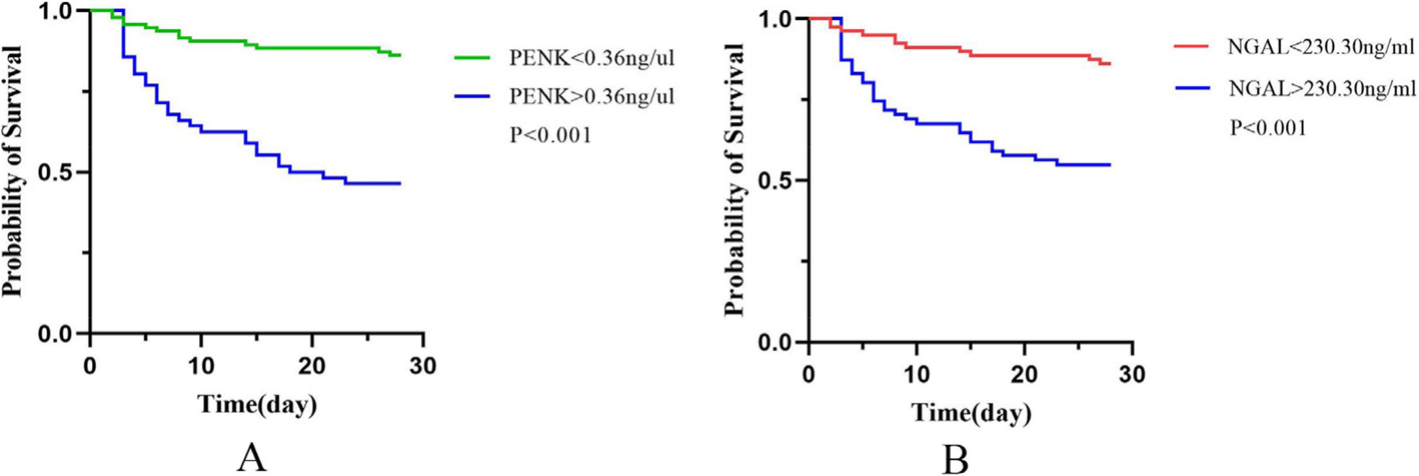
Kaplan-Meier curves for 28-day mortality of p-PENK and p-NAGL

The AUCs were calculated for the 28-day mortality to evaluate predictive capabilities of two biomarkers (Fig. 1, Table 2). In general, the predictive values of p-PENK and p-NGAL for 28-day mortality were good (AUCs 0.785 and 0.700, respectively) (Fig. 1, Table 2). The net reclassification index(NRI) of p-PENK and p-NGAL were 0.22 and 0.21, respectively.The integrated discrimination improvement(IDI) of p-PENK and p-NGAL were both 0.10 (Table 2). However, a combination of the two used together was able to provide even better predictions (AUCs, 0.790). The truncated value of PENK and NGAL for predicting 28-day all-cause mortality was 0.36 ng/µL and 230.30 ng/mL, respectively. The analysis (Table 3) shows that high p-PENK (adjusted HR [aHR] 11.34 [95% CI 3.932–32.703]), p-NGAL (adjusted HR 1.009 [95% CI 1.005–1.013]), age (aHR 1.035 [95% CI 1.009–1.062]), BMI (aHR 0.840 [95% CI 0.724–0.974]), serum albumin levels (aHR 0.929 [95% CI 0.874-0.987]), serum lactate levels (aHR 1.103 [95% CI 1.005–1.211]), and the SOFA score (aHR 1.104 [95% CI 1.043–1.17]) had significant correlations with 28-day mortality (Table 3). Fig. 2 shows Kaplan–Meier curves for 28-day mortality in relation to the p-PENK > 0.36 ng/µL and p-PENK < 0.36 ng/µL groups and to the p-NGAL > 230.30 ng/mL and p-NGAL <2 30.30 ng/mL groups. Values of p-PENK > 0.36 ng/µL and p-NGAL > 230.30 ng/mL were both associated with higher 28-day mortality (Fig. 2).

**Table 2:** Predictive power of p-PENK, p-NGAL, and the combination of p-PENK and p-NGAL for 28-day mortality based on ROC curve analysis

	AUC	95%CI	Sensitivity	Specificity	Youden Index	*p*-value	Cut-off level	NRI^a^	IDI^b^
p-PENK	0.785	0.706-0.865	0.766	0.721	0.487	<0.001	0.36	0.22	0.10
p-NGAL	0.700	0.611-0.789	0.744	0.636	0.380	<0.001	230.30	0.21	0.10
p-PENK+ p-NGAL	0.790	0.712-0.868	0.744	0.710	0.454	<0.001

**Table 3:** Multivariable Cox regression model associated with 28-day mortality

Variable	Hazard ratio	95% CI	*p*-value
Age	1.035	1.009-1.062	0.008
BMI	0.840	0.724-0.974	0.021
Albumin	0.929	0.874-0.987	0.018
Lactate	1.103	1.005-1.211	0.04
SOFA score	1.104	1.043-1.17	0.001
Apache II score	0.997	0.952-1.044	0.898
AKI stage	1.377	0.953-1.99	0.088
p-NGAL	1.009	1.005-1.013	0.001
p-PENK	11.34	3.932-32.703	0.001

## Discussion

In this prospective study, we assessed the predictive value of serum PENK and NGAL levels for 28-day mortality in patients in the ICU with AKI. In addition, we determined optimal serum PENK and NGAL levels. High p‑PENK levels predicted 28-day mortality for patients in the ICU with AKI, with a predictive value of 0.36 ng/µL. High p‑NGAL levels also predicted 28‑day mortality in these patients, with a predictive value of 230.30 ng/mL. Moreover, a combination of the two was even better at predicting 28-day mortality in patients in the ICU with AKI (sensitivity, 0.744; specificity, 0.710).

A meta-analysis of 130 studies found that AKI-related mortality was 23.9% and that at < 3, 3–6, and > 6 months of follow-up, pooled AKI-associated mortality rates were 22.1%, 31.5%, and 27.7%, respectively [11]. In a large multicenter randomized trial, the mortality rate of patients with AKI within 90 days was reported to be 57.6% [12]. Another study of ICU patients showed a slightly lower mortality rate (22% within 28 days [13]. Similarly, we observed a mortality rate of 28.7% within 28 days after ICU admission. These differences may have been because those previous studies mostly focused on sepsis or septic shock, whereas we included patients with all causes of AKI who had been admitted to the ICU.

The association between PENK and AKI has previously been shown in several studies involving patients who were critically ill. [14, 15] The median PENK level increases proportionately with AKI severity [16]. The p-PENK value is highly predictive of short-term mortality and may enable early identification of patients at risk of death. PENK levels have been previously used to predict one-year mortality and transition to CKD [17, 18]. PENK has been shown to predict 30-day mortality in ICU patients [19]. Moreover, some current research has suggested that the PENK threshold level is 80 pmol/L [12]. This may help clinicians identify patients with high probabilities of mortality earlier, which can help them intervene earlier and potentially reduce mortality. In patients with poor outcomes, serum PENK levels were greater than 80 pmol/L. Declines in kidney function may be caused by PENK, through cardiodepression [20, 21]. Proenkephalin A and opioid receptors are widely produced in the body, and are especially abundant in the kidneys [22]. These findings agree with our study results, which showed that patients with high p-PENK levels had a significantly higher risks of mortality. Although the relevant pathophysiology on the subject is currently unclear, this does not prevent the application of p-PENK levels to predict the prognoses of AKI patients, especially in the ICU. In addition, p-PENK level appears to be an independent predictor of kidney complications, as well as cardiovascular and liver-related diseases [23]. Quantified patient p-PENK has been shown to predict postoperative AKI and in-hospital mortality following aortic repair. [24] One study showed that the mortality rates of patients with high PENK levels who did not have AKI was comparable to those of patients with AKI. [25] Recent studies have shown that high PENK levels can predict a worsened prognosis in patients with heart failure [21]. Current literature primarily recognizes PENK for its predictive capabilities regarding AKI occurrence [26–29], and CKD development [30], recovery of kidney function [31], mortality in severe illnesses [17, 19, 27], extended ICU stays and heightened mortality post-cardiac surgery [32], and recovery from neurological dysfunction [33–35]. The novelty of our study lies in demonstrating that plasma PENK not only predicts mortality in ICU patients with AKI but also, when combined with NGAL, significantly enhances mortality prediction in this patient group.

Many studies have shown that serum NGAL can predict acute kidney injury. [36, 37] NGAL protects against kidney damage and prevents renal fibrosis [38]. NGAL can be used for both stratification of AKI and as a guide for the next step in treatment [39]. Serum NGAL levels were elevated in patients who had combined ischemia and infection. This phenomenon might help to explain its high discriminatory capability in relation to the 28-day mortality of patients with AKI in the ICU. P-NGAL is an efficient predictor for the early diagnosis and prognosis of immune-mediated membranous nephropathy (MN) [40]. The level of p-NGAL appears to be an independent predictor of kidney complications, as well as cardiovascular and liver-related diseases [41–43]. Overall, p-NGAL has been shown to be the most promising and clinically available kidney injury biomarker and could be used particularly for patients who are critically ill [43, 44]. Serum p-NGAL has been found to be a good predictor for AKI and a good predictor of the need for renal replacement therapy. In predicting in-hospital mortality, the p-NGAL level showed a statistically significant AUROC of 0.768 in this study. Albumin levels have also shown statistical significance in predicting in-hospital mortality [45]. In our study, the AUROC of p-NGAL showed a similar statistical significance, at 0.768; and low albumin levels, old age, hyperlactic acidemia, body mass index, and SOFA scores were also found to be statistically significant predictors of 28-day mortality. A combination of p-NGAL and p-PENK yielded an AUROC of 0.790 (*P* < 0.001) for in-hospital mortality. Patients who had elevated levels of these biomarkers had higher mortality rates, especially when serum PENK and/or NGAL levels rose [27]. Similarly, in our study, p-PENK of > 0.36 ng/µL and p-NGAL of > 230.30 ng/mL were strongly associated with higher 28-day mortality (Fig. 2). Moreover, the integration of p-NGAL and p-PENK levels was found to further improve the prognoses of patients in the ICU with AKI.

Serum PENK and NGAL levels both show good predictive power for 28-day mortality. In a study of patients discharged from an ICU (*n* = 1,207), higher p-PENK and p-NGAL levels measured at ICU discharge were associated with poor one-year outcomes, including in patients with low serum creatinine levels at ICU discharge [17]. These two biological indicators are considered to be good predictors of mortality in patients with acute kidney injury in the ICU [33]. Our study offers new insight into predicting mortality in patients in the ICU with AKI. There are currently few such studies and more research is needed on the subject. Our study results need to be validated in another cohort of patients in the ICU with AKI.

This study has the following shortcomings: first, this was a small single-center study; second, this study was confined to the ICU; third, this study did not grade AKI; finally, the etiology of AKI was not classified.

In conclusion, serum PENK and NGAL levels in patients in the ICU with AKI showed clinical value for 28-day mortality prediction, at cut-off values of 0.36 ng/µL and 230.30 ng/mL, respectively. A combination of p-PENK and p-NGAL further improved the accuracy of predicting 28-day mortality in patients in the ICU with AKI, while retaining sensitivity and specificity.

## Data Availability

The datasets used and analyzed during the current study are available from the corresponding author upon reasonable request.
